# Ethnic Differences in the Association of Depressive Symptoms with Clinical Outcome in Dialysis Patients

**DOI:** 10.1007/s40615-019-00600-0

**Published:** 2019-06-18

**Authors:** Robbert W. Schouten, Gerlinde L. Haverkamp, Wim L. Loosman, Prataap K. Chandie Shaw, Frans J. van Ittersum, Yves F. C. Smets, Louis-Jean Vleming, Friedo W. Dekker, Adriaan Honig, Carl E. H. Siegert

**Affiliations:** 1 Department of Nephrology, Amsterdam UMC (VU University Amsterdam), Amsterdam, The Netherlands; 2 Department of Psychiatry, Amsterdam UMC (VU University Amsterdam), Amsterdam, The Netherlands; 3 Department of Nephrology, Haaglanden Medical Center, The Hague, The Netherlands; 4 Department of Nephrology, VU Medical Centre, Amsterdam, The Netherlands; 5 Department of Nephrology, Haga Teaching Hospital, The Hague, The Netherlands; 6 Department of Clinical Epidemiology, Leiden University Medical Centre, Leiden, The Netherlands; 7 Department of Psychiatry, Amsterdam Public Health, Amsterdam UMC, Vrije Universiteit Amsterdam, Amsterdam, The Netherlands

**Keywords:** Depression, Mortality, Hospitalization, Dialysis, Interaction, Ethnicity

## Abstract

**Background:**

Studies show mixed results on the association between depressive symptoms and adverse clinical outcomes in patients on dialysis therapy. Ethnicity may play a role in these heterogeneous results. No studies have investigated the interplay between ethnicity and depressive symptoms on clinical outcome in this patient population. This study aims to examine interaction between ethnicity and depressive symptoms on hospitalization and mortality in dialysis patients.

**Methods:**

A multi-ethnic cohort in 10 dialysis centers included 687 dialysis patients between 2012 and 2017, with an average follow-up of 3.2 years. Depressive symptoms were measured using the Beck Depression Inventory. Interaction was assessed by investigating excess risk on an additive scale using both absolute rates and relative risks. Multivariable regression models included demographic, social, and clinical variables.

**Results:**

Adverse outcomes are more pronounced in native patients, compared to immigrant patients. The risk for mortality and hospitalization is considerably higher in native patients compared to immigrants. An excess risk on an additive scale indicates the presence of possible causal interaction.

**Conclusions:**

Depressive symptoms are a risk factor for hospitalization and mortality, especially in native dialysis patients. Adverse clinical events associated with depressive symptoms differ among ethnic groups. This differential association could play a role in the conflicting findings in literature. Ethnicity is an important factor when investigating depressive symptoms and clinical outcome in dialysis patients. Future research should focus on the possible mechanisms and pathways involved in these differential associations.

**Electronic supplementary material:**

The online version of this article (10.1007/s40615-019-00600-0) contains supplementary material, which is available to authorized users.

## Introduction

Depression is the most common psychiatric disorder in patients receiving dialysis therapy. The estimated prevalence of depressive symptoms in dialysis patients is 37% [1]. The burden of depressive symptoms for these patients and society is high with a marked effect on Quality of Life, healthcare costs, and several adverse clinical outcomes. Several large prospective cohort studies showed that depressive symptoms have an association with hospitalizations and all-cause mortality [2–5]. However, studies investigating the association between depressive symptoms and mortality showed mixed results. A recent meta-analysis by Farrokhi et al. [2] showed that only 15 out of 31 included studies reported a significant association. The authors conclude that there is a considerable between-study heterogeneity, without a clear explanation in current literature. One of the factors that might contribute to the heterogeneous results is the ethnic composition of the cohorts.

There is evidence for a differential association between depressive symptoms and adverse clinical outcomes between different ethnic groups [1, 6–11]. Several studies in chronically ill patient populations indicate that ethnicity moderates the relation between depressive symptoms and adverse clinical outcomes [2, 11–15]. Furthermore, psychiatric studies in the general population have shown ethnic differences might play a role in the susceptibility, prevalence, and clinical course of depressive symptoms [1, 16]. Thus, interaction may be present between ethnicity, depressive symptoms, and adverse clinical outcomes.

Although there is data available on survival differences between ethnic groups in dialysis patients, there is limited data available on dialysis patients investigating the possible interaction between depressive symptoms, ethnicity, and adverse clinical outcomes, such as mortality [17]. In addition, most studies investigating depressive symptoms in dialysis patients lack a clear definition for ethnicity, making it difficult to draw conclusions on the effect of ethnicity [2]. To the best of our knowledge, no studies in dialysis patients have been published to investigate the possible interaction between depressive symptoms and ethnicity on adverse clinical outcomes.

This study set out to investigate interaction between ethnicity, depressive symptoms, and adverse clinical outcomes in dialysis patients. Our aim is to describe the differences between the ethnic groups and to provide insight in ethnic differences in the association between depression and adverse clinical outcomes. We hypothesize that a differential association between depressive symptoms and adverse clinical outcomes is present between different ethnic groups. The Depression-In-Various-Ethnicities-and-Races-Study (DIVERS) is a large multi-ethnic prospective dialysis cohort in the Netherlands that has been designed to investigate ethnic differences in depressive symptoms and adverse outcomes.

## Materials and Methods

### Study Cohort

Data were obtained from the DIVERS study (Depression-In-Various-Ethnicities-and-Races-Study). This is an observational, prospective cohort study among chronic dialysis patients in 10 dialysis centers in the Netherlands. The cohort consists of both prevalent and incident dialysis patients, included between 2012 and 2017. All patients who met the inclusion criteria were approached for study participation during dialysis treatment or during an outpatient appointment. Inclusion criteria were at least 18 years of age and a dialysis vintage of at least 90 days. Patients who were unable to fill in self-reported questionnaires were excluded. To improve generalizability, all questionnaires and variables were available in Dutch, English, Turkish, and Moroccan Arabic translations. Before inclusion, all patients gave written informed consent. This study was approved by the medical ethics committees of all participating hospitals and was carried out in accordance with the Helsinki declaration.

### Demographic, Social, and Clinical Data

At baseline, the following socio-demographic and clinical data were collected from electronic medical records: age, gender, dialysis modality and vintage, comorbidities (summarized in the Davies comorbidity score), primary cause of kidney disease, body mass index (BMI), routine laboratory measures, transplantation waiting list, and medication use. Incident dialysis patients were defined as new patients on renal replacement therapy > 90 days but < 180 days. The primary cause of kidney disease was classified according to the European Renal Association-European Dialysis and Transplant Association (ERA-EDTA) coding system and divided into 4 groups (diabetes mellitus, glomerulonephritis, renal vascular disease, and other) [18]. The level of comorbidity was defined according to the Davies comorbidity index, indicating no, intermediate, or severe comorbidity [19].

We collected the following characteristics through self-reported questionnaires: marital status, children, educational level, working status, current smoking and alcohol use, and previous depression. Quality of Life was measured using the Short Form Quality of Life (SF-12). Anxiety symptoms were assessed using the self-questionnaire, the Beck Anxiety Inventory (BAI).

### Definition of Ethnicity

Ethnicity was defined as immigrant status based on country of birth of the patients and of their parents. According to the Statistics Netherlands Criteria, an individual is considered to be an immigrant if the patient himself and/or at least one parent was born abroad [20]. In this manuscript, the term “native” refers to native Dutch patients. Five regions of origin of immigrant patients were distinguished, using the United Nations classification system: Europe, Sub-Saharan Africa, northern Africa/Western Asia, Southern Asia/South Eastern Asia, and South America/Caribbean [21].

### Assessment of Depressive Symptoms

Depressive symptoms were assessed using the Beck Depression Inventory-II (BDI) at baseline, within 2 weeks after enrollment. Respondents were asked to rate how much each of these symptoms bothered them in the past week, on a scale ranging from 0 (not at all) to 3 (severely). The total score has a minimum of 0 and a maximum of 63. The BDI was analyzed primarily using a cutoff value. The BDI has been validated in a large variety of cohorts with various depressive disorders and clinical comorbidities [22]. The BDI has a high internal consistency (Cronbach’s *α* = 0.92) [23, 24]. For the BDI, a cutoff value of 16 has been used to indicate the presence of clinically relevant moderate to severe depressive symptoms [25]. The BDI has been validated in a multi-ethnic Dutch cohort of chronic dialysis patients and showed a good validity with the physician-diagnosed DSM-IV criteria [26]. Previous studies found that the BDI provides an assessment of severity of depressive symptoms that is equivalent across gender, race, and ethnicity in college students [27].

### Clinical Outcome: Hospitalization and Mortality

Cause and time of death and hospitalization data were collected from electronic medical records for a maximum of 4 years. Cause of death was classified using the European Renal Association-European Dialysis and Transplant Association (ERA-EDTA) coding system [18]. Data from baseline to 1 year after inclusion was used in the analysis for the 1-year hospitalization rate.

### Statistical Analysis

The main analysis consists of investigating interaction between ethnicity and depression and their association with mortality in the univariable cox regression model. Secondary analyses include the stepwise multivariable models to investigate possible explanatory variables in this association. Furthermore, the absolute risk differences are described for the groups based on ethnicity and depression as exposure. The 1-year hospitalization rate was the secondary outcome. In addition, the absolute risks hospitalization and death were calculated and compared between ethnic groups.

The first multivariable model includes age and sex. The second multivariable model included sex, age, incident/prevalent, dialysis vintage, dialysis modality, residual diuresis, Davies comorbidity score, diabetes, ischemic heart disease, cancer, albumin, and hemoglobin.

Cox proportional hazard models were used to investigate the univariable and multivariable effect of depressive symptoms on mortality. Time to event was defined as the time between inclusion (baseline) and the date of death or censoring. Patients who either were lost to follow-up, were transplanted, or had recovery of renal function were censored. Poisson regression models were used to study the association between depressive symptoms and hospitalization during the first year after inclusion. Absolute mortality risks were calculated using rates per person-years (number of deaths/1000 person-years).

This study investigates the possibility of causal interaction, which is defined as a deviation from additivity of the absolute effects (risk differences) of the two risk factors under study [28, 29]. The following steps were undertaken to investigate interaction in this cohort:

- Stratification by ethnicity.
- Absolute risk differences of mortality and 1-year hospitalization. Interaction was defined as an excess risk on an additive scale in the absolute risks.
- Relative risk differences: Regression models were used to investigate interaction on a relative scale including possible confounders. All patients were divided into 4 mutually exclusive groups based on depression and ethnicity status. The lowest number was given to the group with the lowest risk on mortality in the cox proportional hazard model.
  - Group 1 (− −): immigrant, no depression.
  - Group 2 (− +): immigrant, depressed.
  - Group 3 (+ −): native Dutch, no depression.
  - Group 4 (+ +): native Dutch, depressed

The relative excess risk due to interaction (RERI) was calculated, which can be interpreted as the risk that is additional to the risk that is expected based on the addition of the risks under exposure. This is calculated as the difference between the expected risk and the observed risk: RERI = (Risk ++) − (Risk +−) − (Risk −+) +1.

The attributable proportion due to interaction (AP) was calculated to investigate the proportion of mortality that is due to interaction among patients who are both depressed and native: AP = RERI/(Risk ++). The synergy index (S) was calculated to interpret the excess risk from exposure to both exposures (depression and native ethnicity) when there is interaction relative to the risk from exposure without interaction: *S* = ((Risk++) − 1)/((Risk+−) − 1) + ((Risk−+) − 1). When there is no interaction effect, the RERI and AP equal 0 and *S* equals 1.

Ninety-five percent confidence intervals of the RERI and SI were calculated using the delta method [30, 31].

As a last step, an interaction term depression*ethnicity will be introduced in the multivariable models as a measure for multiplicative/statistical interaction.

### Missing Values

Baseline demographic and clinical variables had < 5% missing. The overall percentage of missing questions on the BDI is 4.6%. Missing items were analyzed in the complete case analyses using list-wise deletion of missing values. To avoid bias, missing values of the BDI were imputed by using multiple imputation techniques (10 repetitions) as a sensitivity analysis. All statistical analyses were performed using SPSS for Windows version 24.

## Results

### Baseline Characteristics

A total of 687 dialysis patients were included in this multi-ethnic cohort. Table 1 describes the baseline characteristics for all patients and stratified by ethnicity. The cohort consisted of 48% immigrant and 52% native dialysis patients. Compared to native patients, immigrant patients were on average 10 years younger (59 ± 15 versus 69 ± 14 years respectively). Social characteristics were mostly comparable between the 2 groups. Immigrant patients had a higher percentage of diabetic nephropathy compared to native patients. Total comorbidity scores were divided in low (27%), intermediate (55%), and severe (18%) and were comparable between the ethnic groups. Four percent of patients self-reported a previous depression.

**Table 1:** Baseline characteristics of the total cohort and stratified by ethnicity

Characteristic	All patients (*n* 687)	Immigrant (*n* 300, 48%)	Native Dutch (*n* 327, 52%)
Demographic			
Age in years	64 ± 15	59 ± 15	69 ± 13
Sex, % men	424 (62%)	190 (63%)	205 (63%)
Composition of immigrant cohort (WHO regions) - European - Sub-Saharan Africa - Northern Africa/Western Asia - Southern Asia/South-Eastern Asia - South-America/Caribbean	– – – – –	38 (13%) 21 (7%) 54 (18%) 57 (19%) 130 (43%)	– – – – –
Social			
Marital status or living together, % married	316 (52%)	139 (49%)	171 (56%)
Children, % yes	474 (78%)	228 (79%)	234 (77%)
Low education, % highest is primary education	135 (22%)	66 (23%)	68 (16%)
Not employed	534 (89%)	251 (88%)	269 (89%)
Renal and dialysis			
Incident dialysis patients	253 (37%)	98 (33%)	118 (36%)
Prevalent dialysis patients	433 (63%)	202 (67%)	209 (64%)
- Dialysis vintage in months (median, IQR)	12 (4–45)	17 (4–60)	11 (4–42)
Treatment modality: - Hemodialysis - Peritoneal dialysis	601 (88%) 84 (12%)	267 (89%) 33 (11%)	288 (88%) 39 (12%)
Primary renal disease: - Diabetic nephropathy - Renal vascular disease - Glomerulonephritis - Other	155 (24%) 163 (26%) 70 (11%) 247 (39%)	97 (34%) 65 (23%) 29 (10%) 91 (32%)	43 (14%) 84 (28%) 36 (12%) 137 (46%)
Vascular access in HD patients: - Fistula - Graft - Central venous catheter	443 (65%) 65 (10%) 91 (13%)	196 (65%) 32 (11%) 37 (12%)	213 (65%) 30 (9%) 45 (14%)
Kt/V urea at baseline (median, IQR)	2.0 (1.5–3.6)	1.8 (1.4–3.4)	2.0 (1.5–3.5)
Residual diuresis, ≥ 100 ml/24 h	488 (71%)	187 (62%)	2517 (77%)
On waiting list for Tx: - Yes - No, because of medical reasons - No, because of patient preference	203 (29%) 436 (64%) 46 (7%)	119 (40%) 165 (55%) 16 (5%)	71 (22%) 229 (70%) 27 (8%)
Clinical			
Current smoker	108 (18%)	45 (16%)	59 (20%)
Davies comorbidity score: - Low comorbidity - Moderate comorbidity - Severe comorbidity	183 (27%) 370 (55%) 119 (18%)	70 (24%) 172 (59%) 51 (17%)	96 (30%) 165 (51%) 62 (19%)
Comorbidities: - Diabetes mellitus - Chronic heart disease - Peripheral vascular disease	288 (42%) 114 (17%) 84 (12%)	145 (48%) 42 (14%) 35 (12%)	120 (37%) 65 (20%) 47 (14%)
Psychiatric			
Receiving psychological care at baseline	24 (4%)	13 (4%)	11 (4%)
Previous depression	27 (4%)	11 (4%)	14 (4%)
Depressive symptoms: - Mean continuous score - Cutoff ≥ 16	12.9 ± 9.6 163 (31%)	14.5 ± 10.8 97 (39%)	11.2 ± 7.8** 58 (22%)**
Health-related quality of life (SF-12)			
SF-12 physical component mean summary score	38.1 ± 11.1	38.5 ± 11.1	37.8 ± 11.2
SF-12 mental component mean summary score	48.9 ± 10.9	47.1 ± 11.5	50.5 ± 10.0

Only the BDI scores and depression rates (cutoff ≥ 16) between native Dutch and immigrant patients were tested and were *p < 0.05**p < 0.01

The mean BDI score of the total cohort was 12.9 ± 9.6. Immigrant patients had significantly higher BDI scores (mean 14.6 ± 10.9) compared to native patients (mean 11.2 ± 7.8) (*p* < 0.001). Thirty-nine percent of immigrant patients had a BDI of 16 or higher (moderate to severe depressive symptoms), compared to 22% of the native patients. The Quality of Life scores, however, showed no major difference between immigrant and native patients on both the mental and physical component scores. These results show that depressive symptoms are more prevalent among immigrant dialysis patients compared to native dialysis patients while the level of comorbidity is comparable between the groups.

### Association Between Depressive Symptoms and Mortality

Patients were followed up for a maximum of 4 years, with a median of 3.2 years. A total of 172 (26%) patients died during follow-up and 142 patients had a kidney transplantation (21%). Thirty-three (5%) patients were lost to follow-up, including 15 patients who moved to other dialysis centers; 9 patients had a recovery of renal function; and 6 patients were not motivated to continue.

**Fig. 1:**
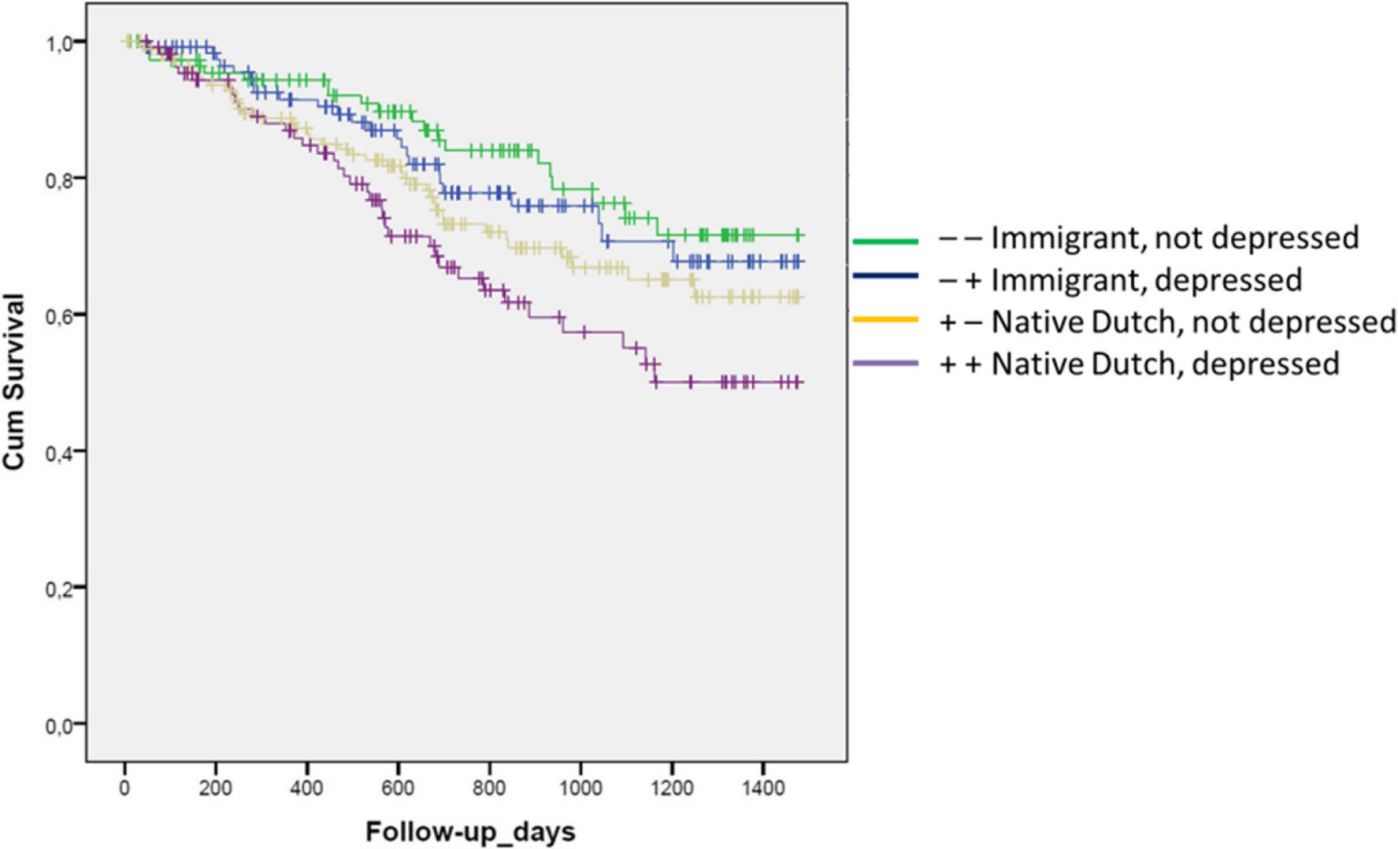
Kaplan–Meier survival plot of groups based on ethnicity and depression

**Fig. 2:**
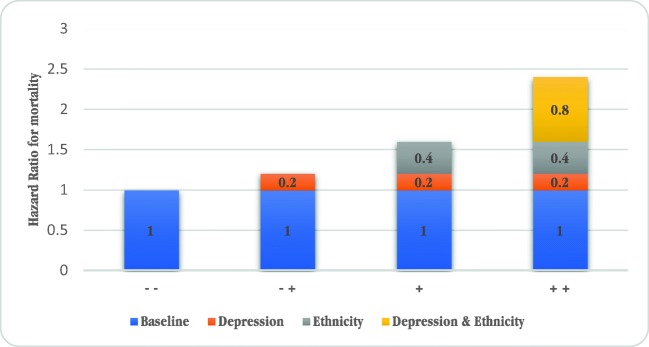
Hazard ratios for mortality with contributions from different exposure categories using model 1

Table 2 shows the HR for mortality by using a grouping variable based on ethnicity and the presence of depressive symptoms. The reference category is the patient group with the lowest mortality risk, the non-depressed immigrant patient group. The HR of depressive symptoms in immigrant patients was 1.2 (0.7–2.1). The non-depressed native patients had a HR for mortality of 1.6 (1.0–2.5) compared to the reference group. The highest risk group was depressed native patients with a HR of 2.4 (1.4–4.2). The survival plot of the different groups based on ethnicity and depression is shown in Fig. 1. The Excess Risk of Interaction (RERI) on an additive scale is 0.64 (95% CI − 0.55–1.83), as visually shown in Fig. 2. The synergy index (SI) was 1.89 (0.47–7.61). These indices show there is evidence for interaction. On a multiplicative scale, the product term depression*ethnicity showed a HR of 1.3 with a *p* value of 0.117. Multivariable models were used to interpret the effect on the hazard ratios after adding possible explanatory variables to the crude model. The multivariable models showed the same trend with a slight decrease in all hazard ratios, with a RERI of 0.47 (− 0.46–1.60) and SI of 2.78 (0.16–47.0) in model 3.

**Table 2:** Hazard ratio for mortality in groups based on ethnicity and depression

Stratification in groups using ethnicity and depression		Hazard ratio for all-cause mortality using stepwise sequential models		
		Model 1: univariable	Model 2: + age, sex	Model 3: + somatic
– –	Immigrant, not depressed (29%)	1.0	1.0	1.0
– +	Immigrant, depressed (20%)	1.2 (0.7–2.1) *p* = 0.6	1.2 (0.7–2.1) *p* = 0.5	1.0 (0.6–1.9) *p* = 0.9
+ −	Native Dutch, not depressed (40%)	1.6 (1.0–2.5) *p* = 0.06	1.2 (0.7–1.8) *p* = 0.7	1.3 (0.8–2.1) *p* = 0.3
+ +	Native Dutch, depressed (11%)	2.4 (1.4–4.2) *p* = 0.003	1.8 (1.0–3.1) *p* = 0.06	1.9 (1.0–3.3) *p* = 0.04

Patients were stratified into 4 mutually exclusive groups based on the presence of depression (BDI ≥ 16) and their ethnicity (immigrant vs native)

The association between depressive symptoms and mortality is investigated using Cox proportional hazard models. Hazard ratios (HRs) are presented including their corresponding 95% confidence interval

To investigate the effect of variables on the association, several stepwise sequential models were used with variables that might be within the causal pathway between ethnicity and mortality

Model 1: univariable/crude model

Model 2: model 1 + age, sex

Model 3: model 2 + incident/prevalent, dialysis vintage, dialysis modality, residual diuresis, Davies, diabetes, ischemic heart disease, cancer, albumin, hemoglobin. Davies comorbidity score includes DM, congestive heart failure, ischemic heart disease, peripheral vascular disease, COPD, liver disease, cancer, and collagen vascular disease

Measure of interaction on additive scale: RERI (95% CI) = 0.64 (− 0.55–1.83), SI = 1.89 (0.47–7.61)

Absolute mortality rates are shown in Table 3. Immigrant patients without depressive symptoms had the lowest mortality rate of 90/1000 person-years. Native patients with depressive symptoms had the highest mortality rate of 211/1000 person-years. For the native patients with depressive symptoms, we expected a rate of 90 (baseline immigrant) + 15 (effect of depressive symptoms in immigrants) + 50 (effect of native Dutch ethnicity) 155/1000 person-years. The observed rate of 211 is higher than 155 by 56/1000 person-years. This leads to an excess risk of 56/1000 person-years on an additive scale.

**Table 3:** Absolute risk of mortality in groups based on ethnicity and depression

Stratification in groups using ethnicity and depression		Absolute mortality rates
– –	Immigrant, not depressed (29%)	90/1000 person-years
– +	Immigrant, depressed (20%)	140/1000 person-years
+ −	Native Dutch, not depressed (40%)	105/1000 person-years
+ +	Native Dutch, depressed (11%)	211/1000 person-years

Absolute risk differences in 1000 person years. These are crude risk differences between immigrant and native Dutch patients

In conclusion, there is evidence for interaction on an additive scale between depressive symptoms and ethnicity related to all-cause mortality, with a more pronounced negative effect of depressive symptoms on mortality in native Dutch patients.

### Association Between Depressive Symptoms and Hospitalization

A total of 359 (52%) patients were admitted to the hospital in the first year after inclusion. The median length of hospital admittance was 6 days (IQR 2–14), and 108 (16%) of the patients had 3 or more hospitalizations per year.

The relative risk of hospitalization from the Poisson regression models is shown in Table 4. All groups are compared with the non-depressed immigrant group as a reference. Depressive symptoms in native Dutch patients show the highest risk for 1-year hospitalization, while depressive symptoms in immigrant patients do not show a significantly higher risk in the fully adjusted model. The RERI is 0.25 (− 0.17–0.67) in the univariable model and 0.45 (0.03–0.86) in model 3. The SI is 1.97 (0.43–9.03) in the univariable model. On a multiplicative scale, the interaction term depression*ethnicity has a *p* value of 0.087. The multivariable models did not show major differences with the univariable model, with a RERI of 0.45 (0.03–0.86) in model 3.

**Table 4:** Relative risks of hospitalization in groups based on ethnicity and depression

Stratification in groups using ethnicity and depression		Rate ratio for hospitalization using stepwise sequential models		
		Model 1: univariable	Model 2: + age, sex	Model 3: + somatic
– –	Immigrant, not depressed (29%)	1.0	1.0	1.0
– +	Immigrant, depressed (20%)	1.3 (1.0–1.6) *p* = 0.04	1.2 (1.0–1.6) *p* = 0.06	1.2 (0.9–1.5) *p* = 0.2
+ −	Native Dutch, not depressed (40%)	1.0 (0.8–1.2) *p* = 0.7	0.8 (0.6–1.0) *p* = 0.06	0.8 (0.7–1.1) *p* = 0.2
+ +	Native Dutch, depressed (11%)	1.5 (1.2–2.0) *p* = 0.003	1.3 (1.0–1.7) *p* = 0.06	1.5 (1.1–1.9) *p* = 0.009

Patients were stratified into 4 mutually exclusive groups based on the presence of depression (BDI ≥ 16) and their ethnicity (immigrant vs native)

The association between depressive symptoms and hospitalization rate is investigated using Poisson regression models. Rate ratios are presented including their corresponding 95% confidence interval

To investigate the effect of variables on the association, several stepwise sequential models were used with variables that might be within the causal pathway between ethnicity and mortality

Model 1: univariable/crude model

Model 2: model 1 + age, sex

Model 3: model 2 + incident/prevalent, dialysis vintage, dialysis modality, residual diuresis, Davies, diabetes, ischemic heart disease, cancer, albumin, hemoglobin. Davies comorbidity score includes DM, congestive heart failure, ischemic heart disease, peripheral vascular disease, COPD, liver disease, cancer, and collagen vascular disease

Measure of interaction on additive scale: RERI (95% CI) = 0.25 (− 0.17–0.65), SI = 1.97 (0.43–9.03)

Absolute 1-year hospitalization rates are shown in Table 5. The lowest rate of 1.1 is shown in the non-depressed immigrant group. When investigating interaction, the depressed native Dutch patients had an excess risk of 0.3 hospitalizations/year.

**Table 5:** Absolute hospitalization rate per year in groups based on ethnicity and depression

Stratification in groups using ethnicity and depression		Hospitalization rate/year
– –	Immigrant, not depressed (29%)	1.1 hospitalizations/year
– +	Immigrant, depressed (20%)	1.1 hospitalizations/year
+ −	Native Dutch, not depressed (40%)	1.4 hospitalizations/year
+ +	Native Dutch, depressed (11%)	1.7 hospitalizations/year

Absolute risk differences in hospitalization rate per year. These are crude risk differences between immigrant and native Dutch patients

### Additional Analyses

Additional stratified analyses show the association between depressive symptoms and adverse clinical outcomes within different ethnic groups, which are shown in Tables 6 and 7. These results showed a larger effect of depressive symptoms on outcome in native Dutch dialysis patients compared to immigrant patients. Furthermore, an analysis using a different definition of ethnicity, Caucasian-Asian-Black, showed no major differences compared to the main analyses, as shown in Supplementary Table S1. Sensitivity analyses using a multiple imputed dataset showed no major differences to the complete case analyses, as shown in supplementary Table S2.

**Table 6:** Stratified analyses of immigrant and native Dutch patients on the association between depression and adverse clinical outcome

Stratified analyses in different ethnic groups	Hazard ratio for all-cause mortality using stepwise sequential models		
	Model 1: univariable	Model 2: + age, sex	Model 3: + somatic
Cohort including all patients	1.2 (0.8–1.7) *p* = 0.4	1.3 (0.9–1.9) *p* = 0.1	1.2 (0.8–1.7) *p* = 0.4
Cohort including immigrant patients only	1.2 (0.7–2.0) *p* = 0.6	1.2 (0.7–2.1) *p* = 0.6	1.0 (0.5–1.8) *p* = 0.98
Cohort including native Dutch only	1.5 (0.9–2.5) *p* = 0.06	1.5 (0.9–2.5) *p* = 0.1	1.5 (0.9–2.5) *p* = 0.1

Three separate analyses were performed using 3 different cohorts. (1) All patients, (2) immigrant only, and (3) native Dutch only

The association between depressive symptoms and mortality is investigated using Cox proportional hazard models. Hazard ratios (HRs) are presented including their corresponding 95% confidence interval

To investigate the effect of variables on the association, several stepwise sequential models were used with variables that might be within the causal pathway between ethnicity and mortality

Model 1: univariable/crude model

Model 2: model 1 + age, sex

Model 3: model 2 + incident/prevalent, dialysis vintage, dialysis modality, residual diuresis, Davies, diabetes, ischemic heart disease, cancer, albumin, hemoglobin. Davies comorbidity score includes DM, congestive heart failure, ischemic heart disease, peripheral vascular disease, COPD, liver disease, cancer, and collagen vascular disease

**Table 7:** Stratified analyses of immigrant and native Dutch patients on the association between depression and adverse clinical outcome

Stratified analyses in different ethnic groups	Rate ratio for hospitalization using stepwise sequential models		
	Model 1: univariable	Model 2: + age, sex	Model 3: + somatic
Cohort including all patients	1.4 (1.2–1.7) *p* < 0.001	1.5 (1.2–1.7) *p* < 0.001	1.4 (1.2–1.7) *p* < 0.001
Cohort including immigrant patients only	1.3 (1.0–1.6) *p* = 0.04	1.3 (1.0–1.6) *p* = 0.07	1.2 (1.0–1.6) *p* = 0.2
Cohort including native Dutch only	1.6 (1.2–2.0) *p* = 0.001	1.6 (1.3–2.1) *p* < 0.001	1.7 (1.3–2.3) *p* < 0.001

Three separate analyses were performed using 3 different cohorts. (1) All patients, (2) immigrant only, and (3) native Dutch only

The association between depressive symptoms and mortality is investigated using Poisson regression models. Rate ratios (RRs) are presented including their corresponding 95% confidence interval

To investigate the effect of variables on the association, several stepwise sequential models were used with variables that might be within the causal pathway between ethnicity and mortality

Model 1: univariable/crude model

Model 2: model 1 + age, sex

Model 3: model 2 + incident/prevalent, dialysis vintage, dialysis modality, residual diuresis, Davies, diabetes, ischemic heart disease, cancer, albumin, hemoglobin. Davies comorbidity score includes DM, congestive heart failure, ischemic heart disease, peripheral vascular disease, COPD, liver disease, cancer, and collagen vascular disease

In conclusion, there is evidence for interaction on an additive scale between depressive symptoms and ethnicity related to all-cause mortality and hospitalization.

## Discussion

This study investigates ethnic differences in the association between depressive symptoms and adverse clinical outcomes. A multi-ethnic cohort of 687 chronic dialysis patients was set up specifically for this area of research. Although depressive symptoms were more frequent in immigrants, the adverse clinical outcomes associated with depressive symptoms were more pronounced in native Dutch patients compared to immigrant patients. Results indicate there is interaction between ethnicity and depressive symptoms in the relation with hospitalization and all-cause mortality. These differential associations could explain part of the heterogeneity in literature on depressive symptoms in dialysis patients.

### Prevalence of Depressive Symptoms in Different Ethnic Groups

This study shows immigrant dialysis patients have a higher prevalence of depressive symptoms compared to native Dutch dialysis patients. These results are comparable with other European studies, which also found a higher prevalence in immigrant patients compared to native Dutch [6, 7, 32, 33]. US studies indicate that Black dialysis patients have a higher prevalence of depressive symptoms compared to White dialysis patients [15, 34, 35]. However, a comparison of our results with US studies is hampered due to the use of other definitions of ethnicity (i.e., Black/White of Hispanic/non-Hispanic). There is evidence that ethnicity itself interferes with self-reported health and self-reported depressive symptoms [36]. For example, a study in immigrant patients showed a difference in symptom domains of their depressive disorder [37]. The findings on ethnic differences in the prevalence of depressive symptoms may be used when implementing screening and psychosocial care in dialysis patients.

### Depressive Symptoms and Adverse Clinical Outcomes

This study confirms the association between depressive symptoms and adverse clinical outcomes in dialysis patients. However, there was a considerable ethnic difference in these associations. Both in hospitalization rate and all-cause mortality, there is a differential association between ethnic groups and interaction seems to be present. These ethnic differences could play a role in the between-study heterogeneity. Available data on ethnic differences suggest that the longitudinal association of depressive symptoms and adverse outcomes is more present in Caucasian patients compared to Black patients, which is in line with our results [11, 38–40]. On the other hand, it was also suggested that depression is more chronic and disabling for Black compared to White patients [38, 41]. However, it is impossible to draw solid conclusions based on the few available studies, since the majority of studies lack a clear definition for ethnicity, and the immigrant-native differences were not investigated in most studies in dialysis patients [3]. For example, only 7 of the 31 studies from the recent meta-analysis from Farrokhi et al. among dialysis patients included ethnicity or race in their adjusted hazard ratios for depression on mortality.

### Mechanism of the Differential Effect Between Ethnic Groups

The interplay between depression, ethnicity, and adverse health outcomes is complex and multifactorial. Several possible mechanisms may play a role in the interaction.

First, cohort studies and registry data have shown that ethnicity influences the prevalence rate of depression. Ethnic minority groups seem to have a higher burden of depressive symptoms in the dialysis population [7, 35].

Second, several possible mechanisms have been proposed between depression and mortality, including the development of intermediate cardiovascular outcomes, parallel inflammatory pathways, involvement of the HPA axis and non-adherence [2, 4, 42–45]. There is evidence that Black patients have a more favorable inflammation and nutritional status that may explain survival advantages [46].

Third, there is evidence for an association between ethnicity and adverse health outcomes [17, 32, 47–49]. Stronks et al. [48] proposed that physical, behavioral, psychosocial, biological, and healthcare use aspects may play a role in the pathway between ethnicity and health outcomes. A study from a nationally representative cohort by Ellis et al. [50] provided support for a differential association between Black and White respondents in depressive symptoms and negative health behavior, such as smoking.

Fourth, the possible causal pathway between ethnicity and outcome is complex, since ethnicity is a variable that is defined at an early stage of life. Thus, most other variables are in the causal pathway between ethnicity and outcome [51].

The multivariable models presented in this manuscript need to be interpreted with these causal pathways in mind. By adding several variables that may well be within the causal pathway between ethnicity and outcome, and looking at the change in effect size, we gain insight in possible explanatory factors. The multivariable models in this study including age, sex, and a large variety of somatic illness markers did not show major differences with the univariable analyses, suggesting that these variables do not play a major role in the pathway.

Despite these hypothesized mechanisms, much is still unknown on the interaction between ethnicity and depression. Future studies are needed to clarify these ethnic differences, including unraveling relevant causal pathways. New studies on depression in dialysis patients should take ethnic differences into account. We want to highlight the importance of ethnically and culturally tailored interventions that may promote depression detection and treatment.

### Strengths and Limitations

Strengths of this study include the large multi-ethnic cohort with balanced numbers of ethnic groups. The follow-up over 3.2 years is substantially longer compared to most other prospective studies in this field of research. The multivariable models include a wide variety of socio-demographic, dialysis-related, clinical, and comorbidity variables to better understand the confounding effects on these complex associations. Our results need to be interpreted with consideration of the following limitations. Despite including many variables in the regression analyses, it is impossible to rule out any unmeasured confounding in these associations. Second, our findings are based on a multi-center sample in the Netherlands and may not be generalizable to populations in other parts of Europe and beyond. Every clinician and researcher should compare their countries’ ethnic composition before generalizing these conclusions. Third, illiterate patients could not be included in this study, creating a possible dropout of this vulnerable patient group. Fourth, although the BDI shows a good validity and consistency in a multi-ethnic cohort in Dutch dialysis patients, no performance measures are available per ethnic group [26]. A study by Whisman et al. [27] in 2013 found that the BDI provides an assessment of severity of depressive symptoms that is equivalent across gender, race, and ethnicity in college students. Thus, research is needed to fully determine the validity of the BDI across ethnic minority groups in medically ill patients, such as dialysis patients. Last, in this field of research, the definition of ethnicity remains difficult, and the objective information such as country of birth does not capture the multidimensional character of ethnicity. In this study, we deliberately use the somewhat imprecise term of ethnicity to define groups based on ancestry (and not on skin color) since this definition may be more suitable for the ethnic minority groups in Europe [52]. Sensitivity analyses using different definitions for ethnicity, such as Caucasian-Asian-Black, showed similar results compared to the main analyses.

## Conclusion

This study showed that depressive symptoms are a risk factor for hospitalization rate and mortality. Adverse clinical events associated with depressive symptoms may differ among ethnic groups, and there is evidence for interaction between ethnicity and depressive symptoms on clinical outcome. These ethnic differences could play a role in the mixed findings in literature. This study shows there is a need to take the ethnic context of each patient into consideration when performing research or screening for depressive symptoms in this population.

## Electronic Supplementary Material

ESM 1 (DOCX 75 kb)

## References

[1] Palmer S, Vecchio M, Craig JC, Tonelli M, Johnson DW, Nicolucci A, Pellegrini F, Saglimbene V, Logroscino G, Fishbane S, Strippoli GFM (2013). Prevalence of depression in chronic kidney disease: systematic review and meta-analysis of observational studies. Kidney Int.

[2] Farrokhi F, Abedi N, Beyene J, Kurdyak P, Jassal SV (2014). Association between depression and mortality in patients receiving long-term dialysis: a systematic review and meta-analysis. Am J Kidney Dis.

[3] Lacson E, Bruce L, Li NC, Mooney A, Maddux FW (2014). Depressive affect and hospitalization risk in incident hemodialysis patients. Clin J Am Soc Nephrol.

[4] Garcia-Llana H, Remor E, Del Peso G, Selgas R (2014). The role of depression, anxiety, stress and adherence to treatment in dialysis patients health-related quality of life: a systematic review of the literature. Nefrologia..

[5] Preljevic VT, Osthus TB, Os I, Sandvik L, Opjordsmoen S, Nordhus IH (2013). Anxiety and depressive disorders in dialysis patients: association to health-related quality of life and mortality. Gen Hosp Psychiatry.

[6] de Wit MA, Tuinebreijer WC, Dekker J, Beekman AJ, Gorissen WH, Schrier AC (2008). Depressive and anxiety disorders in different ethnic groups: a population based study among native Dutch, and Turkish, Moroccan and Surinamese migrants in Amsterdam. Soc Psychiatry Psychiatr Epidemiol.

[7] Haverkamp GL, Torensma B, Vergouwen AC, Honig A (2015). Psychological distress in the hospital setting: a comparison between native Dutch and immigrant patients. PLoS One.

[8] Assari S (2016). Race and ethnic differences in additive and multiplicative effects of depression and anxiety on cardiovascular risk. Int J Prev Med.

[9] Moazen-Zadeh E, Assari S (2016). Depressive symptoms predict major depressive disorder after 15 years among Whites but not Blacks. Front Public Health.

[10] Freudenberger R, Cahn SC, Skotzko C (2004). Influence of age, gender, and race on depression in heart failure patients. J Am Coll Cardiol.

[11] Lewis TT, Guo H, Lunos S, Mendes de Leon CF, Skarupski KA, Evans DA, Everson-Rose SA (2011). Depressive symptoms and cardiovascular mortality in older black and white adults: evidence for a differential association by race. Circ Cardiovasc Qual Outcomes.

[12] Lewis TT, Everson-Rose SA, Colvin A, Matthews K, Bromberger JT, Sutton-Tyrrell K (2009). Interactive effects of race and depressive symptoms on calcification in African American and white women. Psychosom Med.

[13] Loosman WL, Rottier MA, Honig A, Siegert CE (2015). Association of depressive and anxiety symptoms with adverse events in Dutch chronic kidney disease patients: a prospective cohort study. BMC Nephrol.

[14] Assari S, Lankarani MM, Burgard S (2016). Black-white difference in long-term predictive power of self-rated health on all-cause mortality in United States. Ann Epidemiol.

[15] Assari S, Moazen-Zadeh E, Lankarani MM, Micol-Foster V (2016). Race, depressive symptoms, and all-cause mortality in the United States. Front Public Health.

[16] Assari S, Burgard S (2015). Black-White differences in the effect of baseline depressive symptoms on deaths due to renal diseases: 25 year follow up of a nationally representative community sample. J Renal Inj Prev.

[17] van den Beukel TO, Dekker FW, Siegert CE (2008). Increased survival of immigrant compared to native dialysis patients in an urban setting in the Netherlands. Nephrol Dial Transplant.

[18] van Dijk PC, Jager KJ, de Charro F, Collart F, Cornet R, Dekker FW (2001). Renal replacement therapy in Europe: the results of a collaborative effort by the ERA-EDTA registry and six national or regional registries. Nephrol Dial Transplant.

[19] Davies SJ, Phillips L, Naish PF, Russell GI (2002). Quantifying comorbidity in peritoneal dialysis patients and its relationship to other predictors of survival. Nephrol Dial Transplant.

[20] Statistics Netherlands. Someone with a foreign background. Definition used by CBS Statistics Netherlands. 2019. https://www.cbs.nl/engb/dossier/migration-and-integration. Accessed 2019 Apr 20.

[21] Department of Economic and Social Affairs UN. Classification of countries by major area and region of the world. United Nations. 2014. available on http://esa.un.org/wpp/excel-Data/country-Classification.pdf. Accessed May 2019

[22] Wang YP, Gorenstein C (2013). Psychometric properties of the Beck Depression Inventory-II: a comprehensive review. Rev Bras Psiquiatr.

[23] Beck AT, Epstein N, Brown G, Steer RA (1988). An inventory for measuring clinical anxiety: psychometric properties. J Consult Clin Psychol.

[24] Wang YP, Gorenstein C (2013). Assessment of depression in medical patients: a systematic review of the utility of the Beck Depression Inventory-II. Clinics (Sao Paulo).

[25] Beck AT, Steer RA, Brown GK (1996). Manual for the Beck Depression Inventory-II.

[26] Loosman WL, Siegert CE, Korzec A, Honig A (2010). Validity of the Hospital Anxiety and Depression Scale and the Beck Depression Inventory for use in end-stage renal disease patients. Br J Clin Psychol.

[27] Whisman MA, Judd CM, Whiteford NT, Gelhorn HL (2013). Measurement invariance of the Beck Depression Inventory-Second Edition (BDI-II) across gender, race, and ethnicity in college students. Assessment..

[28] de Mutsert R, Jager KJ, Zoccali C, Dekker FW (2009). The effect of joint exposures: examining the presence of interaction. Kidney Int.

[29] Rothman KJ, Greenland S, Walker AM (1980). Concepts of interaction. Am J Epidemiol.

[30] Hosmer DW, Lemeshow S (1992). Confidence interval estimation of interaction. Epidemiology..

[31] Knol MJ, VanderWeele TJ (2012). Recommendations for presenting analyses of effect modification and interaction. Int J Epidemiol.

[32] Haverkamp GL, Loosman WL, Hoekstra T, Dekker FW, Chandie Shaw PK, van den Beukel TO (2016). The association of acculturation and depressive and anxiety symptoms in immigrant chronic dialysis patients. Gen Hosp Psychiatry.

[33] Schrier AC, Hogerzeil SJ, de Wit MA, Beekman AT (2017). Depression and anxiety in Turkish and Moroccan minorities in the Netherlands: prevalence, symptoms, risk factors and protective factors a systematic review. Tijdschr Psychiatr.

[34] Watkins DC, Assari S, Johnson-Lawrence V (2015). Race and ethnic group differences in comorbid major depressive disorder, generalized anxiety disorder, and chronic medical conditions. J Racial Ethn Health Disparities.

[35] Williams DR, Gonzalez HM, Neighbors H, Nesse R, Abelson JM, Sweetman J (2007). Prevalence and distribution of major depressive disorder in African Americans, Caribbean blacks, and non-Hispanic whites: results from the National Survey of American Life. Arch Gen Psychiatry.

[36] Assari S, Dejman M, Neighbors HW (2016). Ethnic differences in separate and additive effects of anxiety and depression on self-rated mental health among Blacks. J Racial Ethn Health Disparities.

[37] Assari S, Moazen-Zadeh E (2016). Ethnic variation in the cross-sectional association between domains of depressive symptoms and clinical depression. Front Psychiatry.

[38] Gonzalez HM, Tarraf W, Whitfield KE, Vega WA (2010). The epidemiology of major depression and ethnicity in the United States. J Psychiatr Res.

[39] Gonzalez HM, Vega WA, Williams DR, Tarraf W, West BT, Neighbors HW (2010). Depression care in the United States: too little for too few. Arch Gen Psychiatry.

[40] Owen WF (1996). Racial differences in incidence, outcome, and quality of life for African-Americans on hemodialysis. Blood Purif.

[41] Assari S, Watkins DC, Caldwell CH (2015). Race attribution modifies the association between daily discrimination and major depressive disorder among Blacks: the role of gender and ethnicity. J Racial Ethn Health Disparities.

[42] Taraz M, Taraz S, Dashti-Khavidaki S (2015). Association between depression and inflammatory/anti-inflammatory cytokines in chronic kidney disease and end-stage renal disease patients: a review of literature. Hemodial Int.

[43] Palmer SC, Vecchio M, Craig JC, Tonelli M, Johnson DW, Nicolucci A, Pellegrini F, Saglimbene V, Logroscino G, Hedayati SS, Strippoli GFM (2013). Association between depression and death in people with CKD: a meta-analysis of cohort studies. Am J Kidney Dis.

[44] Cukor D, Rosenthal DS, Jindal RM, Brown CD, Kimmel PL (2009). Depression is an important contributor to low medication adherence in hemodialyzed patients and transplant recipients. Kidney Int.

[45] Katon WJ (2003). Clinical and health services relationships between major depression, depressive symptoms, and general medical illness. Biol Psychiatry.

[46] Streja E, Kovesdy CP, Molnar MZ, Norris KC, Greenland S, Nissenson AR, Kopple JD, Kalantar-Zadeh K (2011). Role of nutritional status and inflammation in higher survival of African American and Hispanic hemodialysis patients. Am J Kidney Dis.

[47] van den Beukel TO, Verduijn M, le Cessie S, Jager KJ, Boeschoten EW, Krediet RT (2012). The role of psychosocial factors in ethnic differences in survival on dialysis in the Netherlands. Nephrol Dial Transplant.

[48] Stronks K, Snijder MB, Peters RJ, Prins M, Schene AH, Zwinderman AH (2013). Unravelling the impact of ethnicity on health in Europe: the HELIUS study. BMC Public Health.

[49] Yan G, Norris KC, Yu AJ, Ma JZ, Greene T, Yu W, Cheung AK (2013). The relationship of age, race, and ethnicity with survival in dialysis patients. Clin J Am Soc Nephrol.

[50] Ellis EM, Orom H, Giovino GA, Kiviniemi MT (2015). Relations between negative affect and health behaviors by race/ethnicity: differential effects for symptoms of depression and anxiety. Health Psychol.

[51] VanderWeele TJ, Robinson WR (2014). On the causal interpretation of race in regressions adjusting for confounding and mediating variables. Epidemiology..

[52] Stronks K, Kulu-Glasgow I, Agyemang C (2009). The utility of ‘country of birth’ for the classification of ethnic groups in health research: the Dutch experience. Ethn Health.

