# Supplementary material for: Ethnic Differences in the Association of Depressive Symptoms with Clinical Outcome in Dialysis Patients
Source: J Racial Ethn Health Disparities. 2019 Jun 18;6(5):990–1000. doi: 10.1007/s40615-019-00600-0 (PMC6736895; doi:10.1007/s40615-019-00600-0)
Supplement: Supplementary file 1 — (DOCX 75 kb) [file 40615_2019_600_MOESM1_ESM.docx]

**Supplementary tables**

**Analyses using different definitions for ethnicity**

**Definition of ethnicity: Caucasian, Asian, Black**

**Supplementary table S1a. Hazard ratio of mortality in groups based on the presence of depressive symptoms and ethnicity (Caucasian, Asian, Black)**

| *Stratification in groups using ethnicity and depression* | | Hazard Ratio for all-cause mortality using stepwise sequential models | | |
| --- | --- | --- | --- | --- |
|  |  | Model 1: univariable | Model 2: + age, sex | Model 3: + somatic |
| – – – | Black patients, not depressed (11%) | 1.0 | 1.0 | 1.0 |
| – – + | Black patients, depressed (9%) | 1.6 (0.5-4.6) p=0.4 | 1.3 (0.4-3.8) p=0.7 | 1.4 (0.4-4.1) p=0.6 |
| – + – | Asian patients, not depressed (9%) | 1.9 (0.7-5.1) p=0.2 | 1.3 (0.5-3.5) p=0.6 | 1.4 (0.5-3.9) p=0.5 |
| – + + | Asian patients, depressed (12%) | 2.4 (1.0-6.1) p=0.06 | 2.0 (0.8-5.0) p=0.2 | 1.7 (0.7-4.5) p=0.2 |
| + – – | Caucasian patients, not depressed (38%) | 3.0 (1.4-6.5) p=0.006 | 1.7 (0.7-4.0) p=0.2 | 2.3 (1.0-5.3) p=0.05 |
| + – + | Caucasian patients, depressed (21%) | 4.4 (1.9-10.1) p=0.001 | 2.6 (1.1-6.3) p=0.03 | 3.2 (1.3-8.0) p=0.01 |

Patients were stratified into 4 mutually exclusive groups based on the presence of depression (BDI≥16) and their ethnicity (immigrant vs native). The association between depressive symptoms and mortality is investigated using cox proportional hazard models. Hazard Ratios (HR) are presented including their corresponding 95% confidence interval.

To investigate the effect of variables on the association, several models are used with variables that are within the causal pathway between ethnicity and mortality.

- Model 1: Univariable / crude model

- Model 2: Model 1 + age, sex

- Model 3: Model 2 + Incident/prevalent, Dialysis Vintage, Dialysis Modality, Residual diuresis, DAVIES, diabetes, ischemic heart disease, cancer, Albumin, Hemoglobin. DAVIES comorbidity score includes DM, congestive heart failure, ischemic heart disease, peripheral vascular disease, COPD, liver disease, cancer, collagen vascular disease).

**Supplementary table S1a. Relative risks of hospitalization in groups based on the presence of depressive symptoms and ethnicity (Caucasian, Asian, Black)**

| *Stratification in groups using ethnicity and depression* | | Rate Ratio for hospitalization using stepwise sequential models | | |
| --- | --- | --- | --- | --- |
|  |  | Model 1: univariable | Model 2: + age, sex | Model 3: + somatic |
| – – – | Black patients, not depressed (11%) | 1.0 | 1.0 | 1.0 |
| – – + | Black patients, depressed (9%) | 1.4 (1.0-2.1) p=0.08 | 1.3 (0.9-2.0) p=0.1 | 1.4 (0.9-2.1) p=0.1 |
| – + – | Asian patients, not depressed (9%) | 1.2 (0.8-1.7) p=0.3 | 1.2 (0.8-1.7) p=0.3 | 1.1 (0.8-1.6) p=0.6 |
| – + + | Asian patients, depressed (12%) | 1.4 (1.0-1.9) p=0.08 | 1.2 (0.9-1.8) p=0.2 | 1.1 (0.8-1.6) p=0.6 |
| + – – | Caucasian patients, not depressed (38%) | 1.1 (0.8-1.4) p=0.6 | 0.9 (0.6-1.2) p=0.4 | 0.9 (0.7-1.3) p=0.7 |
| + – + | Caucasian patients, depressed (21%) | 1.7 (1.2-2.3) p=0.001 | 1.4 (1.0-2.0) p=0.044 | 1.6 (1.1-2.3) p=0.009 |

Patients were stratified into 4 mutually exclusive groups based on the presence of depression (BDI≥16) and their ethnicity (immigrant vs native). The association between depressive symptoms and hospitalization rate is investigated using Poisson regression models. Rate Ratios are presented including their corresponding 95% confidence interval.

To investigate the effect of variables on the association, several models are used with variables that are within the causal pathway between ethnicity and mortality.

- Model 1: Univariable / crude model

- Model 2: Model 1 + age, sex

- Model 3: Model 2 + Incident/prevalent, Dialysis Vintage, Dialysis Modality, Residual diuresis, DAVIES, diabetes, ischemic heart disease, cancer, Albumin, Hemoglobin. DAVIES comorbidity score includes DM, congestive heart failure, ischemic heart disease, peripheral vascular disease, COPD, liver disease, cancer, collagen vascular disease).

**Definition of ethnicity: White, non-White**

**Supplementary table S1a. Hazard ratio of mortality in groups based on the presence of depressive symptoms and ethnicity (White, non-White)**

| *Stratification in groups using ethnicity and depression* | | Hazard Ratio for all-cause mortality using stepwise sequential models | | |
| --- | --- | --- | --- | --- |
|  |  | Model 1: univariable | Model 2: + age, sex | Model 3: + somatic |
| – – | Non-White patients, not depressed (24%) | 1.0 | 1.0 | 1.0 |
| – + | Non-White patients, depressed (18%) | 1.4 (0.7-2.8) p=0.3 | 1.4 (0.7-2.7) p=0.3 | 1.2 (0.6-2.4) p=0.5 |
| + – | White patients, not depressed (46%) | 2.1 (1.2-3.6) p=0.006 | 1.5 (0.8-2.6) p=0.2 | 1.8 (1.0-3.2) p=0.048 |
| + + | White patients, depressed (13%) | 3.0 (1.6-5.6) p<0.001 | 2.1 (1.1-4.1) p=0.02 | 2.5 (1.3-4.9) p=0.007 |

Patients were stratified into 4 mutually exclusive groups based on the presence of depression (BDI≥16) and their ethnicity (immigrant vs native). The association between depressive symptoms and mortality is investigated using cox proportional hazard models. Hazard Ratios (HR) are presented including their corresponding 95% confidence interval.

To investigate the effect of variables on the association, several models are used with variables that are within the causal pathway between ethnicity and mortality.

- Model 1: Univariable / crude model

- Model 2: Model 1 + age, sex

- Model 3: Model 2 + Incident/prevalent, Dialysis Vintage, Dialysis Modality, Residual diuresis, DAVIES, diabetes, ischemic heart disease, cancer, Albumin, Hemoglobin. DAVIES comorbidity score includes DM, congestive heart failure, ischemic heart disease, peripheral vascular disease, COPD, liver disease, cancer, collagen vascular disease).

**Supplementary table S1b. Relative risks of hospitalization in groups based on the presence of depressive symptoms and ethnicity (White, non-White)**

| *Stratification in groups using ethnicity and depression* | | Rate Ratio for hospitalization using stepwise sequential models | | |
| --- | --- | --- | --- | --- |
|  |  | Model 1: univariable | Model 2: + age, sex | Model 3: + somatic |
| – – | Non-White patients, not depressed (24%) | 1.0 | 1.0 | 1.0 |
| – + | Non-White patients, depressed (18%) | 1.3 (1.0-1.6) p=0.07 | 1.2 (0.9-1.5) p=0.2 | 1.2 (0.9-1.5) p=0.3 |
| + – | White patients, not depressed (46%) | 0.9 (0.8-1.2) p=0.9 | 0.8 (0.6-1.0) p=0.06 | 0.9 (0.7-1.2) p=0.4 |
| + + | White patients, depressed (13%) | 1.6 (1.2-2.0) p=0.001 | 1.3 (1.0-1.7) p=0.06 | 1.5 (1.1-2.0) p=0.006 |

Patients were stratified into 4 mutually exclusive groups based on the presence of depression (BDI≥16) and their ethnicity (immigrant vs native). The association between depressive symptoms and hospitalization rate is investigated using Poisson regression models. Rate Ratios are presented including their corresponding 95% confidence interval.

To investigate the effect of variables on the association, several models are used with variables that are within the causal pathway between ethnicity and mortality.

- Model 1: Univariable / crude model

- Model 2: Model 1 + age, sex

- Model 3: Model 2 + Incident/prevalent, Dialysis Vintage, Dialysis Modality, Residual diuresis, DAVIES, diabetes, ischemic heart disease, cancer, Albumin, Hemoglobin. DAVIES comorbidity score includes DM, congestive heart failure, ischemic heart disease, peripheral vascular disease, COPD, liver disease, cancer, collagen vascular disease).

**Definition of ethnicity: Black, White**

**Supplementary table S1a. Hazard ratio of mortality in groups based on the presence of depressive symptoms and ethnicity (Black, White)**

| *Stratification in groups using ethnicity and depression* | | Hazard Ratio for all-cause mortality using stepwise sequential models | | |
| --- | --- | --- | --- | --- |
|  |  | Model 1: univariable | Model 2: + age, sex | Model 3: + somatic |
| – – | Black patients, not depressed (17%) | 1.0 | 1.0 | 1.0 |
| – + | Black patients, depressed (9%) | 1.5 (0.5-4.6) p=0.4 | 1.2 (0.4-3.7) p=0.7 | 1.3 (0.4-3.9) p=0.7 |
| + – | White patients, not depressed (58%) | 3.0 (1.4-6.5) p=0.006 | 1.6 (0.7-3.8) p=0.3 | 2.1 (0.9-3.9) p=0.1 |
| + + | White patients, depressed (16%) | 4.4 (1.9-10.1) p=0.001 | 2.5 (1.0-6.0) p=0.047 | 3.0 (1.2-7.4) p=0.02 |

Patients were stratified into 4 mutually exclusive groups based on the presence of depression (BDI≥16) and their ethnicity (immigrant vs native). The association between depressive symptoms and mortality is investigated using cox proportional hazard models. Hazard Ratios (HR) are presented including their corresponding 95% confidence interval.

To investigate the effect of variables on the association, several models are used with variables that are within the causal pathway between ethnicity and mortality.

- Model 1: Univariable / crude model

- Model 2: Model 1 + age, sex

- Model 3: Model 2 + Incident/prevalent, Dialysis Vintage, Dialysis Modality, Residual diuresis, DAVIES, diabetes, ischemic heart disease, cancer, Albumin, Hemoglobin. DAVIES comorbidity score includes DM, congestive heart failure, ischemic heart disease, peripheral vascular disease, COPD, liver disease, cancer, collagen vascular disease).

**Supplementary table S1b. Relative risks of hospitalization in groups based on the presence of depressive symptoms and ethnicity (Black, White)**

| *Stratification in groups using ethnicity and depression* | | Rate Ratio for hospitalization using stepwise sequential models | | |
| --- | --- | --- | --- | --- |
|  |  | Model 1: univariable | Model 2: + age, sex | Model 3: + somatic |
| – – | Black patients, not depressed (17%) | 1.0 | 1.0 | 1.0 |
| – + | Black patients, depressed (9%) | 1.4 (1.0-2.1) p=0.08 | 1.3 (0.9-1.9) p=0.2 | 1.4 (0.9-2.1) p=0.1 |
| + – | White patients, not depressed (58%) | 1.1 (0.8-1.4) p=0.6 | 0.8 (0.6-1.2) p=0.3 | 0.9 (0.7-1.3) p=0.7 |
| + + | White patients, depressed (16%) | 1.7 (1.2-2.3) p=0.001 | 1.4 (1.0-2.0) p=0.06 | 1.6 (1.1-2.3) p=0.01 |

Patients were stratified into 4 mutually exclusive groups based on the presence of depression (BDI≥16) and their ethnicity (immigrant vs native). The association between depressive symptoms and hospitalization rate is investigated using Poisson regression models. Rate Ratios are presented including their corresponding 95% confidence interval.

To investigate the effect of variables on the association, several models are used with variables that are within the causal pathway between ethnicity and mortality.

- Model 1: Univariable / crude model

- Model 2: Model 1 + age, sex

- Model 3: Model 2 + Incident/prevalent, Dialysis Vintage, Dialysis Modality, Residual diuresis, DAVIES, diabetes, ischemic heart disease, cancer, Albumin, Hemoglobin. DAVIES comorbidity score includes DM, congestive heart failure, ischemic heart disease, peripheral vascular disease, COPD, liver disease, cancer, collagen vascular disease).

**Supplementary table S2. Hazard ratio of mortality in groups based on the presence of depressive symptoms and ethnicity using imputed data**

| *Stratification in groups using ethnicity and depression* | | Hazard Ratio for all-cause mortality using stepwise sequential models | | |
| --- | --- | --- | --- | --- |
|  |  | Model 1: univariable | Model 2: + age, sex | Model 3: + somatic |
| – – | Immigrant, not depressed (29%) | 1.0 | 1.0 | 1.0 |
| – + | Immigrant, depressed (20%) | 1.3 (0.7-2.2) p=0.4 | 1.2 (0.7-2.1) p=0.4 | 1.1 (0.6-1.9) p=0.7 |
| + – | Native Dutch, not depressed (40%) | 1.7 (1.1-2.6) p=0.01 | 1.2 (0.7-1.8) p=0.5 | 1.3 (0.9-2.1) p=0.2 |
| + + | Native Dutch, depressed (11%) | 2.7 (1.6-4.4) p=<0.001 | 1.9 (1.1-3.1) p=0.02 | 2.1 (1.2-3.5) p=0.008 |

Patients were stratified into 4 mutually exclusive groups based on the presence of depression (BDI≥16) and their ethnicity (immigrant vs native). The association between depressive symptoms and mortality is investigated using cox proportional hazard models. Hazard Ratios (HR) are presented including their corresponding 95% confidence interval.

To investigate the effect of variables on the association, several models are used with variables that are within the causal pathway between ethnicity and mortality.

- Model 1: Univariable / crude model

- Model 2: Model 1 + age, sex

- Model 3: Model 2 + Incident/prevalent, Dialysis Vintage, Dialysis Modality, Residual diuresis, DAVIES, diabetes, ischemic heart disease, cancer, Albumin, Hemoglobin. DAVIES comorbidity score includes DM, congestive heart failure, ischemic heart disease, peripheral vascular disease, COPD, liver disease, cancer, collagen vascular disease).

Measure of interaction on additive scale: RERI (95% CI) = 0.69 (-0.54-1.92), SI 1.71 (0.58-5.04)
